# Low expression of CD200 predicts shorter time-to-treatment in chronic lymphocytic leukemia

**DOI:** 10.18632/oncotarget.6948

**Published:** 2016-01-19

**Authors:** Yi Miao, Lei Fan, Yu-Jie Wu, Yi Xia, Chun Qiao, Yan Wang, Li Wang, Min Hong, Hua-Yuan Zhu, Wei Xu, Jian-Yong Li

**Affiliations:** ^1^ Department of Hematology, The First Affiliated Hospital of Nanjing Medical University, Jiangsu Province Hospital, Nanjing 210029, China; ^2^ Collaborative Innovation Center for Cancer Personalized Medicine, Nanjing Medical University, Nanjing 210029, China

**Keywords:** CD200, mean fluorescence intensity, chronic lymphocytic leukemia, prognosis, time to treatment

## Abstract

CD200, formerly known as OX-2, is a type I glycoprotein that is expressed on a variety of cell types. CD200 has been shown to be overexpressed in chronic lymphocytic leukemia (CLL). Although previous studies have confirmed the diagnostic value of CD200 in differentiating CLL from to other B-cell chronic lymphoproliferative disorders especially mantle cell lymphoma, whether CD200 has prognostic significance in CLL remains to be determined. We evaluated the mean fluorescence intensity (MFI) of CD200 in 307 consecutive, untreated patients with CLL in our center using flow cytometry. Using a CD200 MFI cutoff of 189.5, these cases could be divided into two groups. Patients with lower CD200 MFI (< 189.5) had a significantly shorter time-to-treatment (TTT) than those with higher CD200 MFI (≥ 189.5) (median TTT: 2 months vs 28 months, *p* = 0.0008). However, the effect of CD200 MFI on overall survival was not significant (CD200 MFI < 189.5: undefined vs CD200 MFI ≥ 189.5: undefined, *P* = 0.2379). In subgroup analysis, CD200 MFI retained its prognostic value in patients with favourable characteristics such as Binet stage A disease, mutated IGHV status, normal TP53 or negative CD38 expression. In conclusion, our study identified CD200 MFI as a potential prognostic factor in CLL.

## INTRODUCTION

Chronic lymphocytic leukemia (CLL) is the most prevalent leukemia in adults in western countries. Each year, there are 15,000 new diagnoses and 5,000 CLL deaths in United states [1]. The clinical course of patients with CLL is highly heterogeneous, with some patients dying within short period after presentation, while others have a normal lifespan without any therapy. Rai and Binet staging systems are used to classify patients with CLL, although providing useful information about heterogeneity within the clinical courses of CLL, neither of these two systems is effective in predicting for early disease progression [1]. In the last two decades, a number of clinical and molecular features of prognostic significance, which can be used for risk stratification, have been identified [2]. Serum markers such as soluble CD23, β2-microglobulin or thymidine kinase has proved to be effective prognostic parameters. And for genetic markers of CLL cells, genomic aberrations, gene abnormalities (TP53, ATM), as well as the mutation status of the variable segments of the immunoglobulin heavy chain gene (IGHV) were demonstrated to be predictive of clinical outcome. Besides, CD38 and ZAP70 expression, which can be determined by flow cytometry, were found to correlate with IGHV status, disease progression and survival of patients with CLL [3, 4].

CD200, formerly known as OX-2, is a type I glycoprotein that is expressed by thymocytes, activated T cells, B cells, dendritic cells, endothelial cells, and neurons [5]. CD200 interacts with the receptor for CD200 (CD200R), which is restricted to myeloid-derived antigen presenting cells and a subset of T cells [5]. Through the ligation of CD200R, CD200 delivers an inhibitory signal, leading to the suppression of the T-cell-mediated immune activation [6]. CD200 has both diagnostic and prognostic significance in hematological neoplasms. Several studies have confirmed that CD200 immunophenotyping was of utility in the differential diagnosis of B-cell neoplasms [7–10]. CD200 was uniformly expressed on CLL leukemic cells while, in mantle cell lymphoma (MCL) cases, CD200 was only expressed in a minority of CD5 positive cells in a small subset of patients and totally absent in most cases, suggesting its value in the differential diagnosis between CLL and MCL [7]. Besides, hairy cell leukemia (HCL) samples showed bright CD200 expression in a homogenous pattern, indicating that CD200 may help differentiate HCL from other CD5 negative mature B cell neoplasms [11].

Given the value of CD200 in the diagnosis of CLL has been well established, the prognostic role of this marker in CLL remains to be determined. Although previous studies did not establish an association between CD200 expression and outcomes in patients with CLL, the sizes of samples of these studies were relatively small-scale [12, 13]. In this study, we investigate the prognostic value of CD200 expression in a relative large cohort of patients with CLL.

## RESULTS

### Patient characteristics

**Figure 1:**
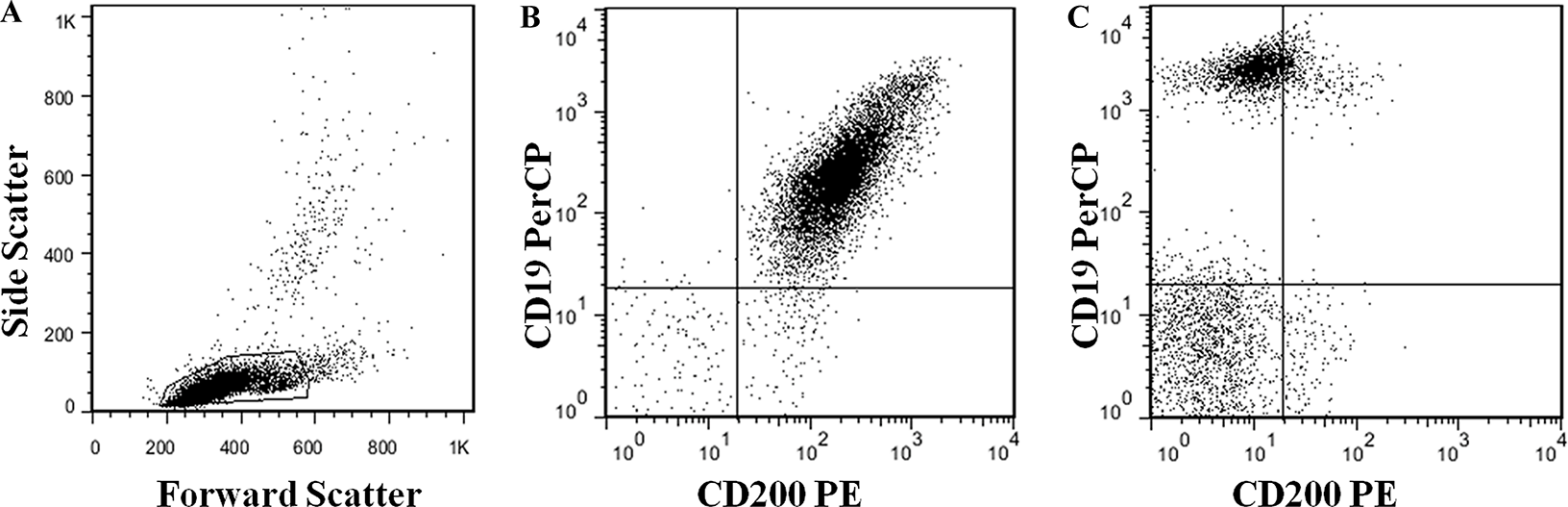
Gating information (A) and representative data plots of CD200 positive (B) and negative (C) cases

**Figure 2:**
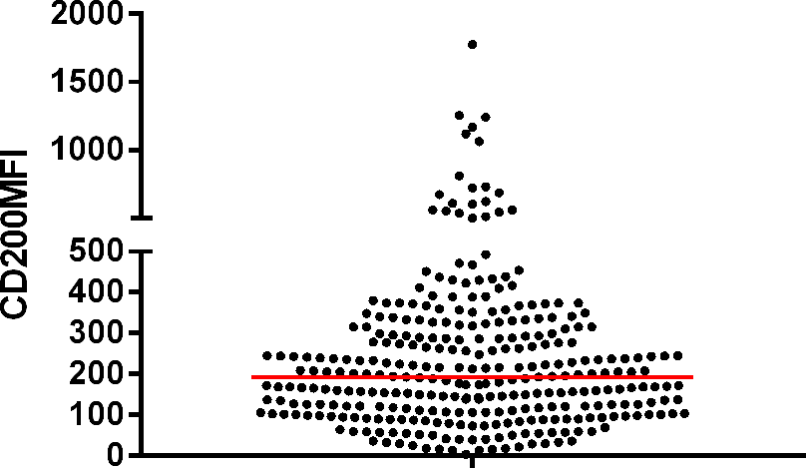
CD200 MFI of CD19+CD200+ cells in CLL cases, the red line indicated the median CD200 MFI

We studied 307 patients with CLL. Clinical characteristics of these patients are summarized in Table 1. The male-to-female ratio was 1.98:1, and the median age was 61 year. Binet stages of 284 patients were available, and of these patients, 110 patients had stage A, 80 had stage B, and 94 had stage C disease. The percentages of CD200 positive cells ranged from 1.52% to 100% (median: 98.8%), and when using 30% as a cutoff for CD200 positivity, only 7/307 (2.3%) patients were difined as negative for CD200 expression. 278/307 (90.6%) patients had CD200 expression in more than 90% CD19 positive cells. The representative data plots of CD200 negative and positive cases were shown as Figure 1A–1C. The median CD200 MFI for all the samples was 192.6 (range: 3.1 to 1777) (Figure 2). Median follow-up is 51 months (range, 12–202 months). Median TTT was 11 months (range, 1–164 months), and median survival was 38 months (range, 1–182 months). 190/307 (61.9%) patients have been treated during follow-up, 9/307 (2.9%) patients had transformed to Richter's syndrome (RS), and 35/307 (11.4%) patients were dead.

**Table 1:** Clinical characteristics of CLL patients at diagnosis

Clinical Characteristics at CLL diagnosis		
Age (years) ^a^	61	53–69
Male	203/307	66.1%
Binet stage		
A	110/307	35.8%
B	80/307	26.1%
C	94/307	30.6%
Peripheral blood lymphocytes (10^9^ per liter)	20.0	10.0–43.8
Hb (g/100 ml)	12.6	10.4–13.8
Platelets (10^9^ per liter)	134	97–179
β2-microglobulin (mg/L)	3.1	2.4–3.5

Abbreviation: CLL, chronic lymphocytic leukemia

a Median and 25th-75th percentiles are reported for continuous variables

### Survival analysis

**Figure 3:**
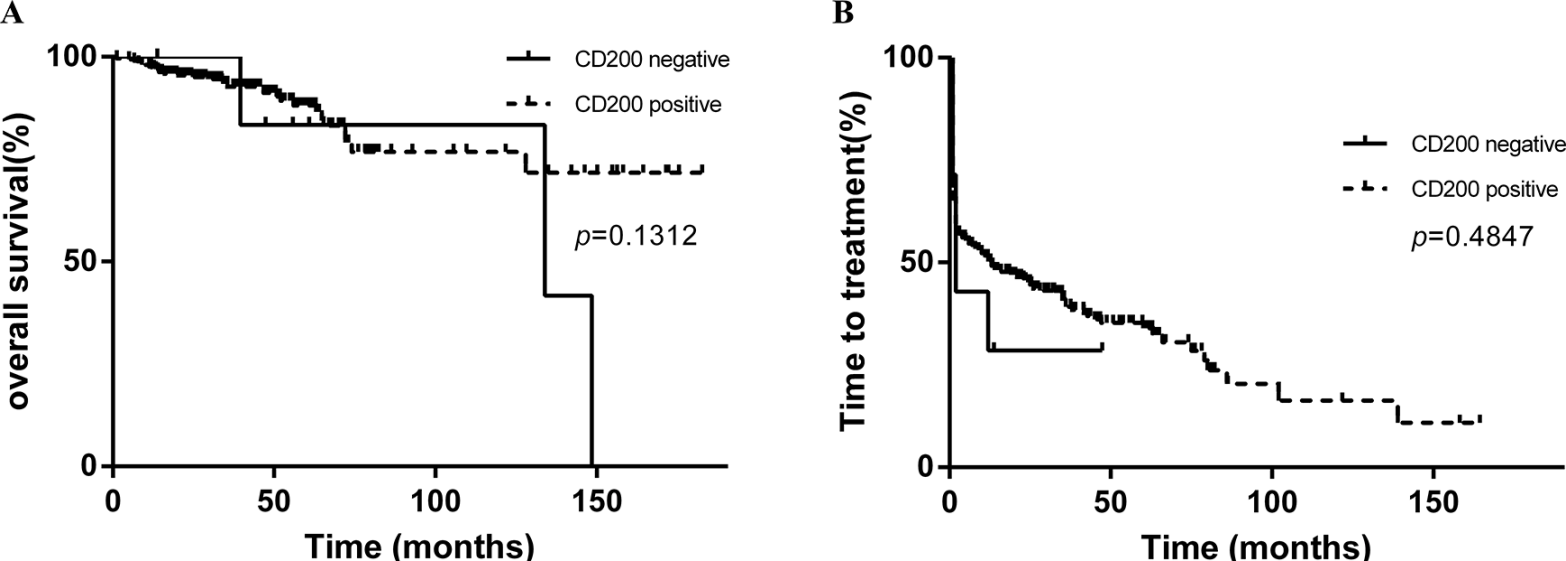
Kaplan-Meier curves of TTT and survival based on CD200 expression (positive versus negative) There was no significant difference between CD200 positive cases and negative cases in OS (**A**) and TTT (**B**).

**Figure 4:**
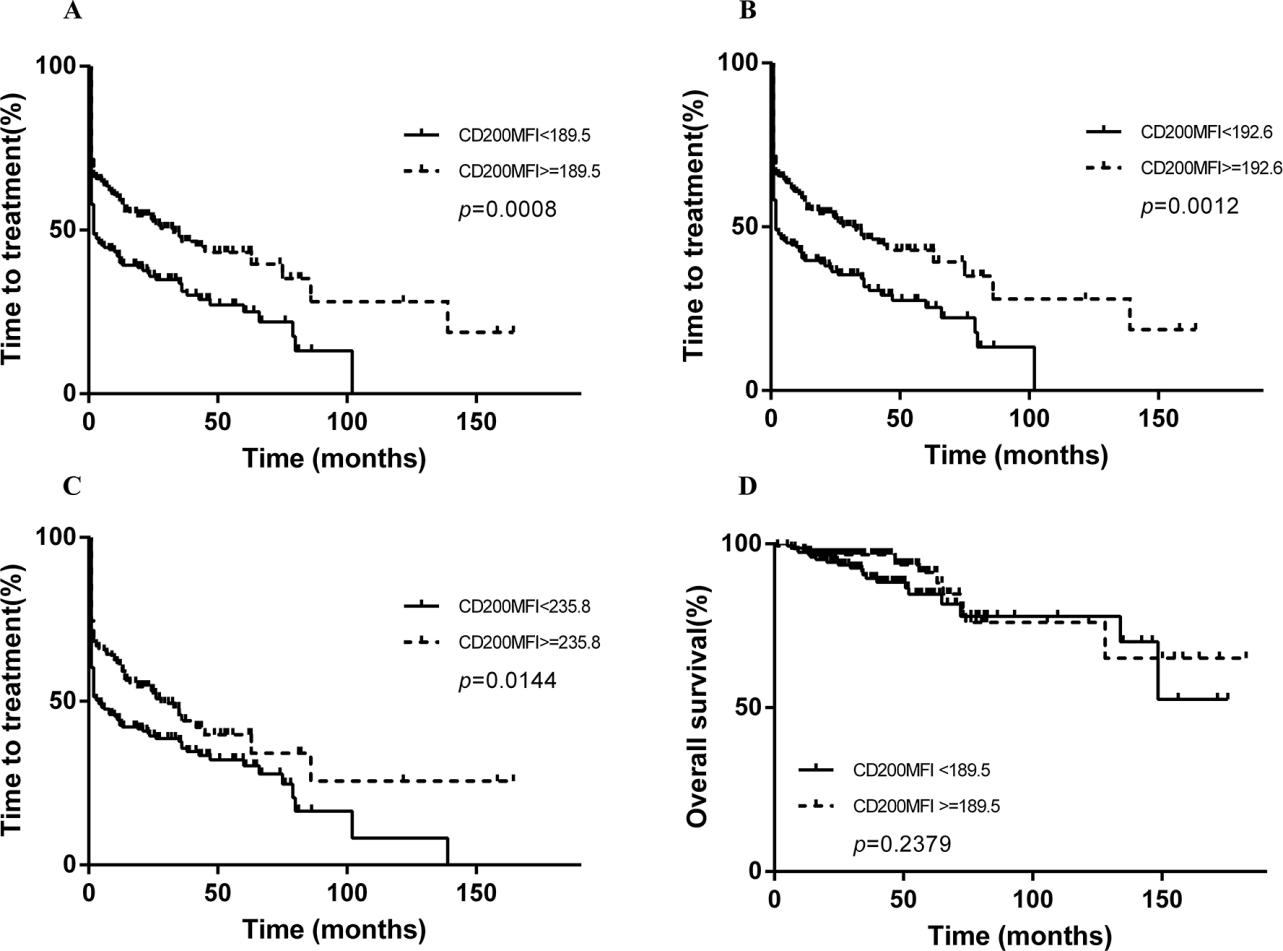
Kaplan-Meier curves of TTT and survival based on CD200 MFI CD200 MFI < 189.5 was predictive of reduced TTT (**A**), by using median CD200 MFI 192.6 as cutoff, the median TTT for patients with low CD200 MFI and high CD200 MFI were 2 months and 33 months, respectively (**B**), while using mean CD200 MFI 235.8 as cutoff, the median TTT for patients with low CD200 MFI and high CD200 MFI were 4 months and 28 months, respectively (**C**), CD200 MFI < 189.5 was not predictive of reduced survival (**D**).

**Figure 5:**
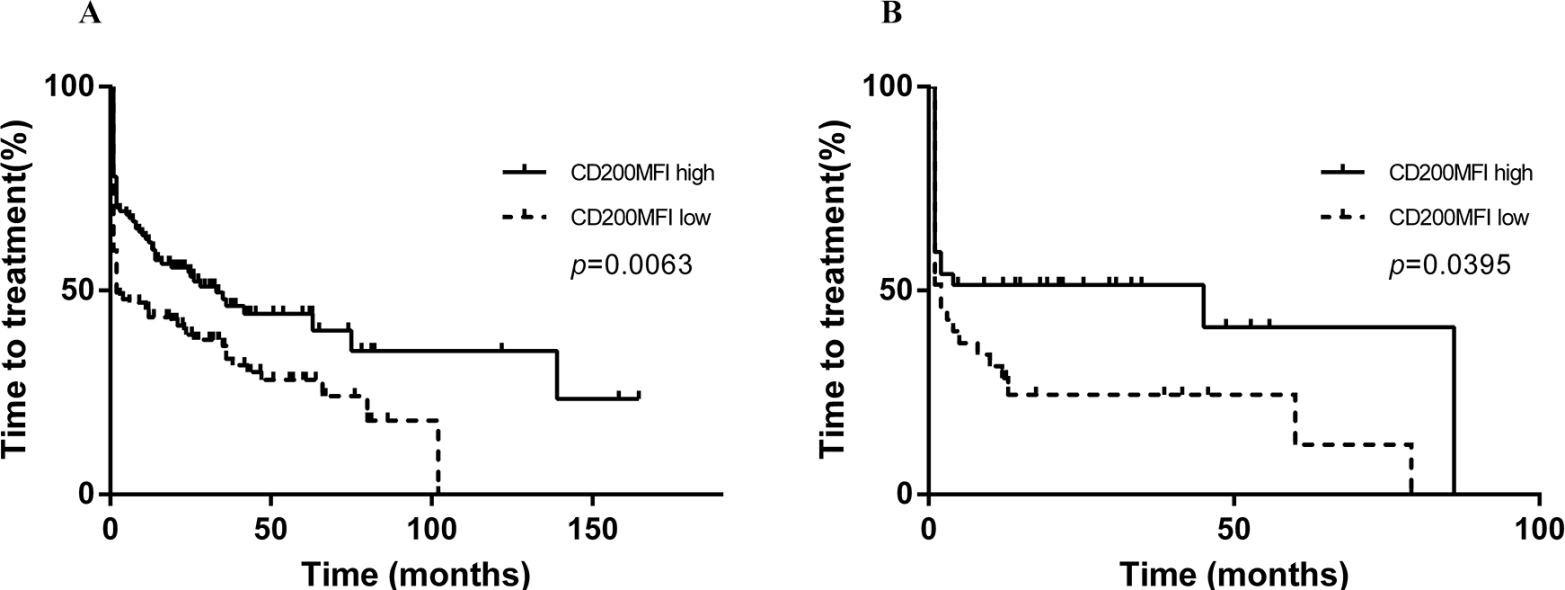
CD200 MFI < 189.5 was also predictive of shorter TTT in 235 patients with PB specimens (A) and 72 patients with BM specimens (B)

Only seven patients in our cohort have a negative CD200 expression when the cutoff value for positive CD200 expression was 30%. There was no significant difference in overall survival (OS) or TTT between positive group and the pure negative group (OS undetermined vs 133.9 months, *p* = 0.1312; TTT 13 months vs 2 months, *p* = 0.4847, Figure 3A, 3B). Then we aimed to determine a CD200 cutoff that best predicted the outcome of CLL patients. While using death as state variable, ROC analysis failed to find a cutoff value that was predictive of death events (AUC = 0.5378, *p* = 0.4615). Subsequently, we tried to find a cutoff of CD200 MFI that was capable of predicting short TTT. In this study, we arbitrarily defined “TTT less than 24 months” as “short TTT”. In ROC analysis, patients who need treatment within 2 years from diagnosis were defined as high-risk group (166 patients), while patient who were free from treatment more than 2 years were included as control group (98 patients). ROC analysis showed that high-risk group could be differentiated from control group (AUC = 0.5934, *p* = 0.01130), and the maximum Youden index was 0.191. Accordingly, the best CD200 MFI cutoff is 189.5. Based on this cutoff, these cases could be divided into two groups with 152/307 (49.5%) patients harboring CD200 MFI < 189.5. Patients with lower CD200 MFI (< 189.5) had a significantly shorter TTT than those with higher CD200 MFI (> 189.5) (median TTT: 2 months vs 30 months, *p* = 0.0008, Figure 4A). Similar results were achieved when using median CD200 MFI or mean CD200 MFI as cut-off value (Figure 4B, 4C). However, the effect of CD200 MFI on overall survival was not significant (CD200 MFI < 189.5: undefined vs CD200 MFI ≥ 189.5: undefined, *p* = 0.2379, Figure 4D). CD200 MFI < 189.5 also predicted lower TTT in 235 patients with PB specimens (Figure 5A) and 72 patients with BM specimens (Figure 5B).

### Univariate and multivariate cox regression analysis of TTT

CD200 MFI, Binet stage, IGHV status, TP53 status, CD38 expression and ZAP70 expression were included in univariate analysis. In univariate analysis, lower CD200 MFI, Binet B/C stage, unmutated IGHV status, TP53 aberration as well as CD38 expression were significant in predicting shorter TTT (Table 2). Subsequently, these five parameters were included in multivariate Cox regression analysis. And in the multivariable Cox model, Binet B/C stage was identified as an independent predictor of reduced TTT (Hazards ratio (HR) 4.552; 95% CI 2.862–7.239; *p* < 0.0001), alongside with unmutated IGHV status (HR 1.489; 95% CI 1.057–2.098; *p* = 0.0228) and TP53 aberration (HR 1.592; 95% CI 1.094–2.315; *p* = 0.0150). However, CD200 MFI < 189.5 was not an independent predictor (HR 1.288; 95% CI 0.924–1.795; *p* = 0.1359), indicating that its significance in predicting reduced TTT might be dependent on its correlation with other factors.

**Table 2:** Univariate and multivariate Cox regression analysis of TTT

Variables	Univariate analysis		Multivariate analysis	
	HR (95% CI)	*P* value	HR (95% CI)	*P* value
CD200MFI < 189.5	1.549 (1.161–2.067)	0.0028	1.288 (0.924–1.795)	0.1359
Binet B/C	4.954 (3.357–7.312)	< 0.0001	4.552 (2.862–7.239)	< 0.0001
Unmutated IGVH	1.866 (1.374–2.534)	< 0.0001	1.489 (1.057–2.098)	0.0228
TP53 disruption	1.982 (1.410–2.787)	< 0.0001	1.592 (1.094–2.315)	0.0150
CD38 positive	1.569 (1.115–2.285)	0.015	1.270 (0.845–1.909)	0.2503

Abbreviations: HR, Hazards Ratio; 95% CI, 95% confidence interval; MFI, mean fluorescence intensity.

### Association between CD200 MFI and clinical, cytogenetic and molecular characteristics of CLL patients

It should be mentioned that we then analyzed CD200 MFI as a binary variable. Interestingly, in this cohort, CLL with CD200 MFI < 189.5 showed a male predominance (*p* = 0.0037, Table 3). In addition, at CLL diagnosis, CD200 MFI < 189.5 was associated with advanced clinical stage (Binet B/C, *p* = 0.0390). Moreover, CD200 MFI < 189.5 was associated with the presence of a positive direct or indirect antiglobulin test (*p* = 0.0223). Additionally, no correlation was found between CD200 MFI < 189.5 and presence of cytogenetic aberrations including del (11q22–q23), del (13q14) or +12 (Table 3). Del (17p13) and TP53 mutation, which are powerful prognostic factors in CLL, were defined as “TP53 aberrations” in our study [14]. And CD200 MFI < 189.5 was not associated with TP53 aberrations in CLL. Among other classical molecular prognostic markers, CD200 MFI < 189.5 was not associated with unmutated IGHV status, CD38 expression or ZAP70 expression. We then examined the association of CD200 MFI < 189.5 with newly reported MYD88, NOTCH1 and SF3B1 mutations, we did not include BIRC3 mutation in our study because of its low incidence among patients in our center [15]. Interestingly, cases with CD200 MFI < 189.5 were more likely to harbor MYD88 mutation than cases with CD200 MFI > 189.5 (*p* = 0.0031), but not NOTCH 1 or SF3B1 mutation (Table 3). In cases with CD200 MFI < 198.5, 7/152 (4.6%) patients transformed to Richter syndrome (RS), while 2/155 (1.3%) patients transformed to RS in cases with CD200 MFI ≥ 198.5 (odds ratio: 3.70, 95% CI: 0.85–18.08, *p* = 0.1015).

**Table 3:** Association between CD200MFI < 189.5 between clinical, cytogenetic and molecular characteristics of CLL patients

	CD200MFI < 189.5	CD200MFI ≥ 189.5	*P* value		CD200MFI < 189.5	CD200MFI ≥ 189.5	*P* value
Male	113/152 (74.3%)	90/155 (58.1%)	0.0037	CD38 ≥ 30%	27 (18.9%)	20 (13.1%)	0.2036
Female	39/152 (25.7%)	65/155 (41.9%)		CD38 < 30%	116 (81.1%)	133 (86.7%)	
≥ 60	83/151 (55.0%)	81/155 (52.3%)	0.6842	ZAP70 ≥ 20%	52 (43.0%)	69 (50.4%)	0.2615
< 60	68/151 (45.0%)	74/155 (47.7%)		ZAP70 < 20%	69 (57.0%)	68 (49.6%)	
A	46/141 (32.6%)	64/143 (44.8%)	0.0390	IGHV mutated	49 (38.9%)	57 (40.7%)	0.8026
B + C	95/141 (67.4%)	79/143 (55.2%)		IGHV unmutated	77 (61.1%)	83 (59.3%)	
DAG or IAT positive	19/85 (22.4%)	9 (9.2%)	0.0223	TP53 mutation or deletion	29 (21.1%)	26 (18.3%)	0.4534
DAG and IAT both negative	66 (76.4%)	89 (91.8%)		without TP53 aberration	102 (77.9%)	116 (81.7%)	
+ 12	17 (17.7%)	19 (18.6%)	1.0000	MYD88 mutated	13 (15.3%)	3 (2.9%)	0.0031
without + 12	79 (82.3%)	83 (81.4%)		MYD88 wild-type	72 (84.7%)	100 (97.1%)	
11q–	13 (14.8%)	9 (9.4%)	0.3633	Notch1 mutated	4 (4.7%)	10 (9.6%)	0.2667
without 11q–	75 (85.2%)	87 (90.6%)		Notch1 wild-type	82 (95.3%)	94 (90.4%)	
13q–	21 (33.3%)	27 (35.1%)	0.8596	SF3B11 mutated	2 (2.2%)	6 (5.7%)	0.2890
without 13q–	42 (66.7%)	50 (64.9%)		SF3B11 wild-type	90 (97.8%)	100 (94.3%)	

Abbreviations: DAT: direct antiglobulin test; IAT: indirect antiglobulin test.

### Dynamic expression of CD200 during progression

**Figure 6:**
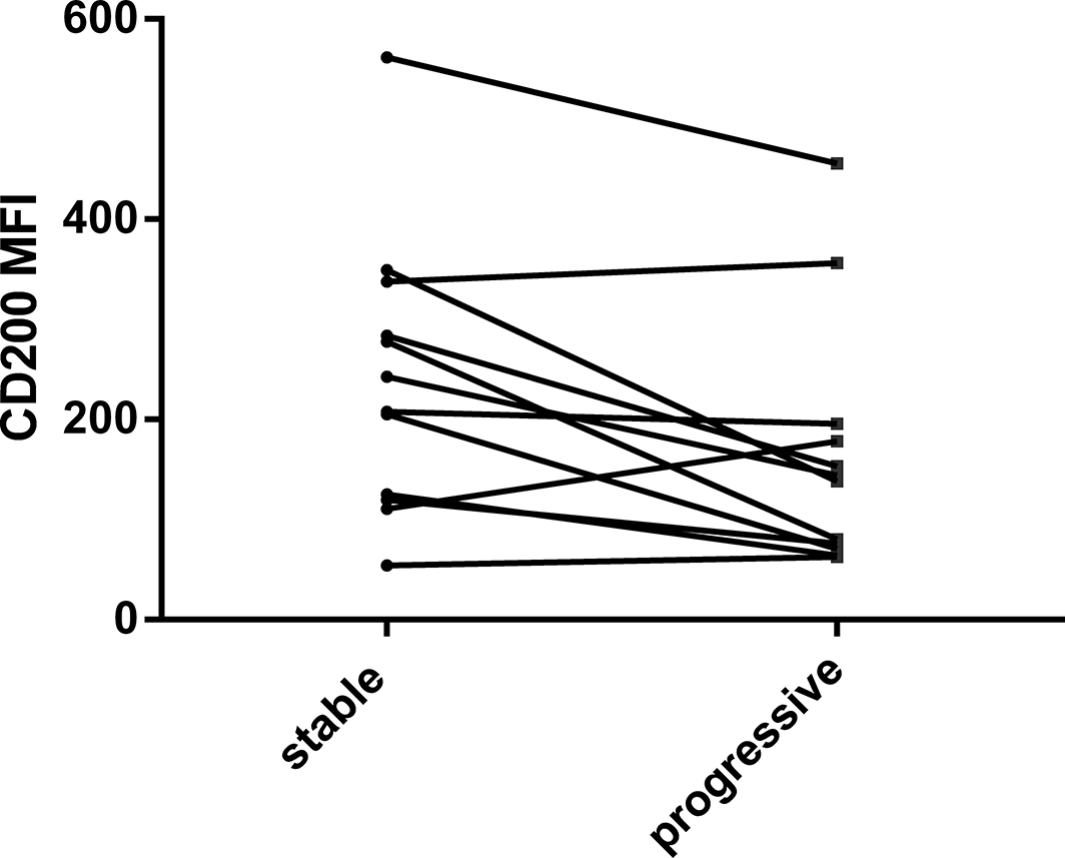
Changes in CD200 MFI during progression in 11 cases

Sequential samples over periods ranging 4.6 months to 46.8 months were harvested from 10 patients who experienced progression (Table 4). In these 10 patients, 8 patients progressed to an advanced Binet stage. For patient 6, the disease was thought to be progressive because lymphocyte count doubling time was less than 6 months. And for patient 10, progressive lymphoadenophathy indicated the progression of disease. For these patients, the first sample was taken in stable disease while the second sample was taken in progressive disease state. Wilcoxon matched-pairs signed rank test demonstrated that CD200 expression decrease when patients progressed (median: stable state 209.8 vs progressive state 141.0, *p* = 0.0323, Figure 6). And in Case 2, 7 and 8, the decrease in CD200 MFI was accompanied by increase in CD38 expression.

**Table 4:** Changes in CD200 MFI during progression

Patient Number	CD200 MFI (s)	CD38 expression (s)	Binet Stage (s)	CD200 MFI (*p*)	CD38 expression (*p*)	Binet Stage (*p*)	Time Interval (months)
1	110.6	negative	A	178.4	negative	C	41.3
2	119.8	negative	B	76.4	32.6%	C	46.8
3	54.3	negative	B	62.9	negative	C	12.6
4	207.8	negative	A	195.9	negative	C	22.1
5	242.6	negative	A	144	negative	C	36.1
6	125.3	negative	B	64.4	NA	B	16.5
7	277.8	55.1%	A	80.4	89.3%	C	26.4
8	283.6	negative	A	153	78%	C	25.7
9	561.8	negative	A	455.9	negative	B	4.6
10	337.8	NA	B	356.2	55.4%	B	5.0
11	205.3	NA	A	70.7	negative	C	8.3

Abbreviations: s indicated stable, p indicated progressive, NA: not available.

### CD200 improves the risk stratification of CLL subgroups identified by conventional prognostic factors

**Figure 7:**
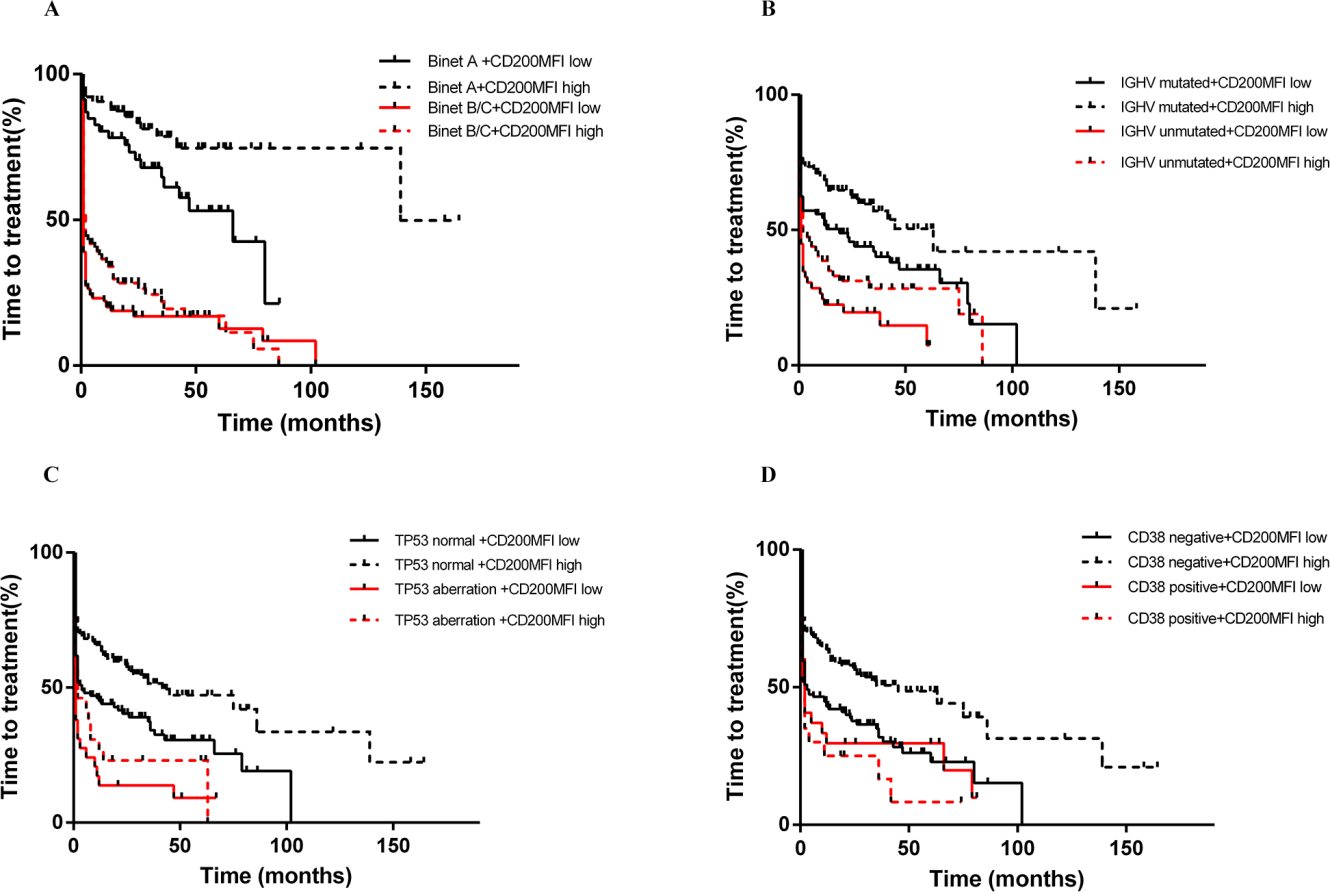
CD200 MFI < 189.5 refined TTT classified by Binet stage (A), IGHV status (B), TP53 status (C) and CD38 expression (D)

In fact, a sizeable proportion of patients in this cohort showed CD200 MFI < 189.5 in absence of unfavorable prognostic factors (Binet B/C stage, unmutated IGHV, TP53 aberrations, CD38 ≥ 30%, ZAP70 ≥ 20%, Table 3). We did not analyze the prognostic value of CD200 MFI in subgroups identified by ZAP70, since ZAP70 ≥ 20% did not predict reduced TTT. CD200 MFI < 189.5 was still capable of segregating a group of patients displaying relatively short TTT in CLL patients with early stage disease (Binet A), mutated IGHV status, normal TP53 or CD38 < 30% (Figure 7). In 110 Binet A stage CLL patients, median TTT was 66 months for patients with CD200 MFI < 189.5 and 139 months for patients with CD200 MFI ≥ 189.5 (*p* = 0.0215, Figure 7A). In 160 patients with mutated IGHV status, median TTT was 19 months for patients with CD200 MFI < 189.5 and 63 months for patients with CD200 MFI (*p* = 0.0190, Figure 7B). In 218 patients with normal TP53 status, median TTT was 4 months for patients with CD200 MFI < 189.5 and 42 months for patients with CD200 MFI ≥ 189.5 (*p* = 0.0032, Figure 7C). In 249 patients without CD38 expression, median TTT was 3 months for patients with CD200 MFI < 189.5 and 45 months for patients with CD200 MFI ≥ 189.5 (*p* = 0.0005, Figure 7D). In addition, CD200 MFI < 189.5 tended to predict reduced TTT in patients with unmutated IGHV status (CD200 MFI < 189.5: median, 1 month vs CD200 MFI ≥ 189.5: median, 2 months), although without significance (*p* = 0.0623).

**Figure 8:**
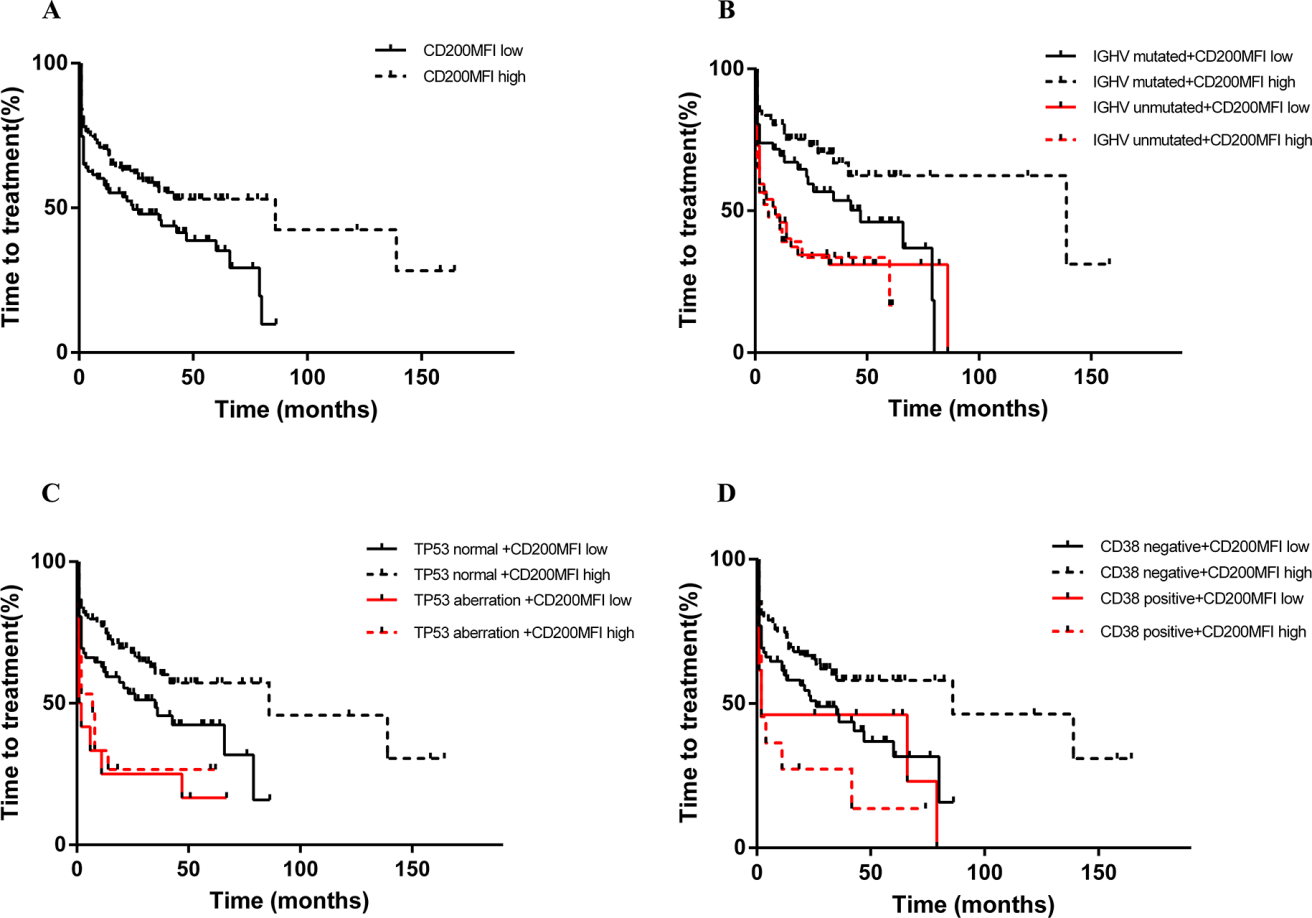
CD200 MFI < 189.5 predicted shorted TTT in patients with Binet A/B diseases (A), and also improved prognostification by IGHV status (B), TP53 status (C), and CD38 expression (D)

**Figure 9:**
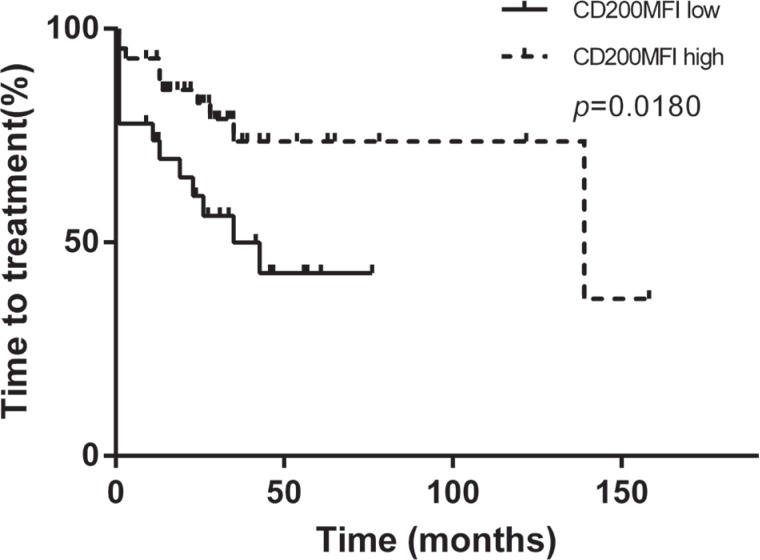
CD200 MFI < 189.5 indentified patients with relatively rapid progression in 70 patients in Binet A/B stage without any classical unfavorable prognostic factor (TP53 aberration, unmuated IGHV status, or CD38 expression)

As patients in Binet C stage usually need immediate treatment, We also separately analyzed the prognostic value in patients in Binet A/B stage. CD200 MFI < 189.5 predicted still predicted shorter TTT (24 months in lower group vs 86 months in higher group, *p* = 0.0244, Figure 8A). In patients in Binet A/B stage, CD200 MFI < 189.5 could identify patients with relatively short TTT in CLL patients with mutated IGHV status, normal TP53 or CD38 < 30%. In 107 patients with mutated IGHV status in Binet A/B stage, median TTT was 47 months for patients with CD200 MFI < 189.5 and 139 months for patients with CD200 MFI (*p* = 0.0443, Figure 8B). In 146 patients with normal TP53 status in Binet A/B stage, median TTT was 35 months for patients with CD200 MFI < 189.5 and 86 months for patients with CD200 MFI ≥ 189.5 (*p* = 0.0343, Figure 8C). In 160 patients without CD38 expression in Binet A/B stage, median TTT was 26 months for patients with CD200 MFI < 189.5 and 86 months for patients with CD200 MFI ≥ 189.5 (*p* = 0.0202, Figure 8D). We also analyzed the prognostic significance of CD200 MFI in 70 patients in Binet A/B stage without any classical unfavorable prognostic factor (TP53 aberration, unmuated IGHV status, or CD38 expression), median TTT was 35 months for patients with CD200 MFI < 189.5 and 139 months for patients with CD200 MFI ≥ 189.5 (*p* = 0.0180, Figure 9).

## DISCUSSION

The diagnostic utility of CD200 in CLL has been investigated in several studies [7]. Although the prognostic relevance of CD200 in CLL has been investigated in previous studies, the prognostic value of CD200 in CLL remains elusive. In the study by McWhirter et al. no correlations were found between CD200 expression and other prognostic markers including CD38 and ZAP70 [13]. In another study by Challagundla et al. the association between CD200 MFI and cytogenetic abnormality was studied, which revealed that +12 tended to show dimmer CD200 expression [12]. However, these studies were carried out on relatively small samples and the authors only determined the association between CD200 expression and other prognostic factors, while the impact of CD200 expression on survival or TTT was not included.

We provided the largest available study of the prognostic role of CD200 in CLL. And in our study, although CD200 expression was uniformly observed in CLL, MFI of CD200 staining varied widely in our cohort, which was similar to a previous study [16]. Using a MFI cutoff of 189.5, we found that the low intensity of CD200 expression predicted worse outcome in patients with CLL. The prognostic significance was also confirmed while using other cutoffs including median and mean CD200 MFI. However, multivariate Cox regression analysis indicated that CD200 MFI was not an independent predictor of worse outcome. We further analyzed the relationship between CD200 MFI and clinical, cytogenetic and molecular features. CD200 MFI < 189.5 was associated with advanced clinical stage (Binet B/C), which indicated that the enrichment of advanced diseases in patients with CD200 MFI < 189.5 might explain the reduced TTT in these patients. Of note, CD200 MFI < 189.5 was shown to retain its prognostic value in Binet A stage CLL patients, suggesting CD200 MFI < 189.5 as a significant risk factor in patients with early-stage disease. Interestingly, CD200 MFI < 189.5 was significantly associated with MYD88 mutation, a newly reported mutation that was associated with favorable outcome. It is worth to mention that CLL patients with MYD88 mutation had a higher frequency of mutated IGHV and low expression of CD38 and ZAP-70 [17]. These results indicated that CLL patients with MYD88 mutation might harbor unique immunophenotypes (low CD200 MFI, low CD38 and ZAP70 expression). Interestingly, CD200 MFI < 189.5 showed a male predominance, however the reasons for this phenomenon were not clear. Further experimental studies may be helpful to elucidate the underlying mechanisms. The rate of RS was higher in patients with lower CD200 MFI; however, this difference was not statically significant. Longer follow-up is needed to confirm this result. Furthermore, CD200 MFI refined the prognostication based on conventional CLL risk factors, which suggested that addition of CD200 MFI to classical prognostic factors should improve the risk stratification in patients with CLL. It should be noted that, in this cohort, we included both PB and BM specimens in our study. In the study by Challagundla et al. paired BM/PB flow cytometry analysis revealed that there was a significant difference in CD200 MFI between specimens involving BM and PB (median, 9,334 vs 5,975; *P* = .002) [12]. In this regard, it is reasonable to consider whether the specimen is from PB or BM while interpreting the result of CD200 expression. However, in our cohort, CD200 MFI retained its prognostic value in PB samples and BM samples, suggesting CD200 MFI < 189.5 may be applicable to both PB samples and BM samples in predicting shorter TTT.

It should be noted that in our cohort only 7 cases were CD200 negative, as a result, We evaluate the prognostic value of the intensity of CD200 expression in CLL. However, MFI, which is used to determine the intensity of CD200 expression, is instrument-dependent. For example, in the study by Challagundla et al. although the same antibody conjugate anti-CD200 PE (clone number: MRC OX-104), the median CD200 MFI was 6444, which is much higher than that in our study [12]. Therefore, our conclusion that CD200 MFI < 189.5 predicted shorter TTT in CLL may be not applicable to other laboratories, and they should have their own CD200 MFI cutoff to predict the outcome of CLL patients although a training set might be needed to determine the cutoff value.

Suppression of patients' T cells has long been implicated in the pathogenesis of CLL [18]. Previous studies demonstrated that up-regulation of CD200 on CLL was sufficient to inhibit Th1 response, including cytokines such as IFN-g and IL-2. And CD200 blockade by monoclonal antibody resulted in restoration of Th1 cytokine profile [13]. Furthermore, CD200 expression on CLL has been engaged in the induction of regulatory T cells, suggesting that CD200 blockade may promote antigen specific-T cell response through suppression of regulatory T cells, which have an immune suppressive role in the initiation and progression of neoplasms [19].

CD200 expression as a prognostic factor has been studied in multiple myeloma (MM). However the results of several studies are controversial [6]. Moreaux et al. reported that patients with CD200 absent MM cells had a better event-free survival compared with those with CD200 positive MM cells [6]. But this finding seems to be inconsistent with another study in which CD200 expression was shown to be inversely correlated with the 70-gene signature, suggesting that loss of CD200 expression may be associated with more aggressive disease [20]. Besides, in a recent study, CD200 expression in plasma cell myeloma (PCM) was found to be associated with lower β2-microglobulin [21]. Additionally, for acute myeloid leukemia, CD200 expression was significantly associated with worse survival [22]. It could be concluded that the prognostic role of CD200 expression appears to be diverse in hematological neoplasms.

Given the immune inhibitory role of CD200 expression in CLL, and from this perspective, it could be postulated that low CD200 may confer a better prognosis. In contrast, our study revealed that lower CD200 expression was associated with worse outcome. There are several explanations for this observation: first, CD200 expression is commonly hypothesized to play a causal role in promoting CLL development via the suppression of antitumor immune responses. However, depending on the context, it is also possible that CD200 inhibits CLL progression by dampening inflammation, which is thought to promote tumor progression in many cancers [23]. Second, it is possible that CD200 plays no major functional roles in modulating CLL progression, and lower CD200 MFI may correlate with another factor that plays a role in determining poor prognosis, as observed in MM. [20] One previous report showed that high soluble CD200 (sCD200) correlated with advanced clinical stage and elevated β2-microglobulin in CLL and sCD200 was critical for engraft CLL cells in immunocompromised mice. Interestingly, ADAM (a disintegrin and metalloproteinase) enzymes, which are involved in membrane protein shedding, contributed to higher plasma sCD200 levels in CLL [24]. It is likely that the shedding of CD200 molecule from the cell membrane may result in low surface CD200 levels in CLL. However, this assumption need to be examined in further studies. Third, there is a possibility that low CD200 MFI is associated with CLL of a particular stage, as observed in our study, Binet B/C stage. Also, our data revealed CD200 MFI decreased when patients progressed to advanced stages, further supporting this hypothesis. More studies are warranted to confirm these hypotheses and find other potential mechanisms.

Finally, CD200 MFI emerged as a potential flow-cytometry based marker that is predictive of TTT. Although markers including IGHV status or P53 status are independent predictors of TTT, technically, their detection is relatively complex compared to CD200. Moreover, CD200 is also an efficient marker in differentiating CLL from MCL, which make it superior to the flow-cytometry based marker CD38 [7–10]. More importantly, in patients in Binet A/B stage without any classical unfavorable prognostic factor (TP53 aberration, unmuated IGHV status, or CD38 expression), lower CD200 MFI identified a subgroup of patients with more rapid progression, indicating that CD200 is a reliable supplementary marker to classical prognostic factors.

In conclusion, our study established CD200 as a marker of both diagnostic [7–10] and prognostic values, which should be included in routine CLL antibody panel.

## METHODS

### Patients

Between November 2009 and January 2015, 307 fresh peripheral blood (PB)/bone marrow (BM) samples were collected from consecutive, unselected patients of CLL. There were 240 PB samples and 67 BM samples in our study. In our cohort, 70 patients were diagnosed prior to 2009. Diagnosis of CLL was established based on criteria of the International Workshop on CLL-National Cancer Institute (IWCLL-NCI). [25] Informed consents were provided according to the Declaration of Helsinki. All the samples were collected prior to treatment.

### Analyses of MYD88, NOTCH1, SF3B1 and TP53 mutations and IGHV mutations

DNA isolation and Sanger sequencing of MYD88, NOTCH1, SF3B1 and TP53 were preformed as previously described [15]. IGHV sequencing was performed as described and germline IGHV was defined as ≥ 98% germline homology.

### Cytogenetics

Interphase fluorescence *in situ* hybridization (FISH) analysis for del (13q14), +12, del (11q22.3) and del (17p13) was performed using standard protocols [14]. Fluorescent-labeled probes included: LSI D13S319 for detection of del (13q14), LSI ATM for detection of del (11q22.3), CEP12 (centromere 12) for detection of +12 and LSI p53 for detection of del (17p13). Cutoff levels for positivity were 10%, 7.7%, 3.0% and 5.2% for del (13q14), del (11q22.3), +12 and del (17p13), respectively.

### Immunophenotyping

The following antibody conjugates were used: anti-CD19-Peridinin-Chlorophyll-Protein Complex (PerCP), anti-CD5-allophycocyanin (APC), anti-CD23-phycoerythrin (PE), FMC7-fluorescein isothiocyanate (FITC), anti-CD148-PE, anti-CD22-FITC, anti-CD20-PE, anti-CD45-PerCP, anti-CD38-APC, anti-surface immunoglobulin (sIg) κ-FITC, anti-sIgλ-PE, anti-CD16-FITC, anti-CD56-FITC, anti-CD3-FITC and anti-ZAP-70-PE. All the antibodies were from BD biosciences (San Jose, CA) and the anti-CD200 clone was MRC OX-104. Flow cytometry analysis was performed on fresh PB/BM specimen. Fresh whole blood samples were incubated with a mixture of fluorescence labeled antibodies for 15 min at room temperature in the dark. Mature red blood cells were lysed using Tris-NH4Cl solution. Four color immunofluorescence were used as follows: FMC7/CD23/CD19/CD5, CD22/CD20/CD45/CD38, sIgκ/sIgλ/CD19/CD45 and CD3-CD16-CD56/ZAP-70/CD19/CD5. Other combinations including CD19/CD200 and CD19/CD148 were used to determine CD200 and CD148 expression, respectively. Isotype matched negative controls were used in all assays to determine positive from negative results. All immunophenotyping of this study were performed on a FACScalibur flow cytometer (Becton-Dickinson, USA) which was equipped with red and blue lasers. Routine machine calibration was made daily according to the standard operating procedure of our laboratory. Fluorescence compensation calibration was run at least once a week using standard fluorescence beads (Calibrite beads, Becton-Dickinson). Data analysis was carried out using CellQuest software, lymphocytes were delineated using forward scatter/side scatter dot plots. CD19 positive cells then were gated. B cells with CD200 expression were analyzed within CD19 positive lymphocytes. In this study, the level of CD200 expression was indicated by the mean fluorescence intensity (MFI). Both the percentage of CD200 positive B cells and CD200 MFI were analyzed. Cases with more than 30% CD19 positive cells expressing CD200 were defined as CD200 positive cases. Immunophenotyping of CD38 and ZAP70 were performed as previously reported [26]. Cutoff points of 30% and 20% were used to define positivity for CD38 and ZAP70, respectively.

### Statistical analysis

The Mann-Whitney U test was used for comparison of continuous variables between subgroups of CLL patients. Wilcoxon matched-pairs signed rank test was used to test the difference between paired samples. Categorical variables were compared by χ^2^-test or Fisher's exact test. Survival was defined as time from diagnosis until death or last follow-up. Time-to-treatment (TTT) was calculated as time from diagnosis until first treatment. ROC (receiver operating characteristic curve) and AUC (area under the ROC curve) was applied to find a cutoff of CD200 MFI that best predicted death or short TTT. Youden index was calculated, the Youden index combines information on sensitivity and specificity to give an overall evaluation of the percentage gain in certainty of predicting worse outcome [27]. Survival curves were plotted using Kaplan-Meier method and log-rank test was used for comparison. Multivariate analysis was performed by multivariate Cox model. All statistical analyses were performed using Graphpad Prism 6 (GraphPad Software, San Diego, CA), SPSS (version 19.0) software (IBM Corporation, Armonk, NY, USA) and Microsoft Office 2010 software for Windows. Statistical significance was defined as *P* value less than 0.05.

## References

[1] Nabhan C, Rosen ST (2014). Chronic lymphocytic leukemia: a clinical review. Jama.

[2] Montillo M, Hamblin T, Hallek M, Montserrat E, Morra E (2005). Chronic lymphocytic leukemia: novel prognostic factors and their relevance for risk-adapted therapeutic strategies. Haematologica.

[3] Damle RN, Wasil T, Fais F, Ghiotto F, Valetto A, Allen SL, Buchbinder A, Budman D, Dittmar K, Kolitz J, Lichtman SM, Schulman P, Vinciguerra VP (1999). Ig V gene mutation status and CD38 expression as novel prognostic indicators in chronic lymphocytic leukemia. Blood.

[4] Crespo M, Bosch F, Villamor N, Bellosillo B, Colomer D, Rozman M, Marce S, Lopez-Guillermo A, Campo E, Montserrat E (2003). ZAP-70 expression as a surrogate for immunoglobulin-variable-region mutations in chronic lymphocytic leukemia. The New England journal of medicine.

[5] Barclay AN, Wright GJ, Brooke G, Brown MH (2002). CD200 and membrane protein interactions in the control of myeloid cells. Trends Immunol.

[6] Moreaux J, Hose D, Reme T, Jourdan E, Hundemer M, Legouffe E, Moine P, Bourin P, Moos M, Corre J, Mohler T, De Vos J, Rossi JF (2006). CD200 is a new prognostic factor in multiple myeloma. Blood.

[7] Palumbo GA, Parrinello N, Fargione G, Cardillo K, Chiarenza A, Berretta S, Conticello C, Villari L, Di Raimondo F (2009). CD200 expression may help in differential diagnosis between mantle cell lymphoma and B-cell chronic lymphocytic leukemia. Leukemia research.

[8] Sandes AF, de Lourdes Chauffaille M, Oliveira CR, Maekawa Y, Tamashiro N, Takao TT, Ritter EC, Rizzatti EG (2014). CD200 has an important role in the differential diagnosis of mature B-cell neoplasms by multiparameter flow cytometry. Cytometry Part B, Clinical cytometry.

[9] Fan L, Miao Y, Wu YJ, Wang Y, Guo R, Wang L, Shen AL, Chen YY, Xu W, Li JY (2015). Expression patterns of CD200 and CD148 in leukemic B-cell chronic lymphoproliferative disorders and their potential value in differential diagnosis. Leukemia & lymphoma.

[10] El Desoukey NA, Afify RA, Amin DG, Mohammed RF (2012). CD200 expression in B-cell chronic lymphoproliferative disorders. Journal of investigative medicine.

[11] Brunetti L, Di Noto R, Abate G, Gorrese M, Gravetti A, Raia M, Scalia G, Pascariello C, Camera A, Del Vecchio L (2009). CD200/OX2, a cell surface molecule with immuno-regulatory function, is consistently expressed on hairy cell leukaemia neoplastic cells. British journal of haematology.

[12] Challagundla P, Medeiros LJ, Kanagal-Shamanna R, Miranda RN, Jorgensen JL (2014). Differential expression of CD200 in B-cell neoplasms by flow cytometry can assist in diagnosis, subclassification, and bone marrow staging. American journal of clinical pathology.

[13] McWhirter JR, Kretz-Rommel A, Saven A, Maruyama T, Potter KN, Mockridge CI, Ravey EP, Qin F, Bowdish KS (2006). Antibodies selected from combinatorial libraries block a tumor antigen that plays a key role in immunomodulation. Proceedings of the National Academy of Sciences of the United States of America.

[14] Dong HJ, Zhou LT, Zhu DX, Wang DM, Fang C, Zhu HY, Zhuang Y, Miao KR, Xu W, Li JY (2011). The prognostic significance of TP53 mutations in Chinese patients with chronic lymphocytic leukemia is independent of del (17p13). Annals of hematology.

[15] Xia Y, Fan L, Wang L, Gale RP, Wang M, Tian T, Wu W, Yu L, Chen YY, Xu W, Li JY (2015). Frequencies of SF3B1, NOTCH1, MYD88, BIRC3 and IGHV mutations and TP53 disruptions in Chinese with chronic lymphocytic leukemia: disparities with Europeans. Oncotarget.

[16] Wong KK, Khatri I, Shaha S, Spaner DE, Gorczynski RM (2010). The role of CD200 in immunity to B cell lymphoma. Journal of leukocyte biology.

[17] Martinez-Trillos A, Pinyol M, Navarro A, Aymerich M, Jares P, Juan M, Rozman M, Colomer D, Delgado J, Gine E, Gonzalez-Diaz M, Hernandez-Rivas JM, Colado E (2014). Mutations in TLR/MYD88 pathway identify a subset of young chronic lymphocytic leukemia patients with favorable outcome. Blood.

[18] Gorgun G, Holderried TA, Zahrieh D, Neuberg D, Gribben JG (2005). Chronic lymphocytic leukemia cells induce changes in gene expression of CD4 and CD8 T cells. The Journal of clinical investigation.

[19] Pallasch CP, Ulbrich S, Brinker R, Hallek M, Uger RA, Wendtner CM (2009). Disruption of T cell suppression in chronic lymphocytic leukemia by CD200 blockade. Leukemia research.

[20] Alapat D, Coviello-Malle J, Owens R, Qu P, Barlogie B, Shaughnessy JD, Lorsbach RB (2012). Diagnostic usefulness and prognostic impact of CD200 expression in lymphoid malignancies and plasma cell myeloma. American journal of clinical pathology.

[21] Douds JJ, Long DJ, Kim AS, Li S (2014). Diagnostic and prognostic significance of CD200 expression and its stability in plasma cell myeloma. Journal of clinical pathology.

[22] Tonks A, Hills R, White P, Rosie B, Mills KI, Burnett AK, Darley RL (2007). CD200 as a prognostic factor in acute myeloid leukaemia. Leukemia.

[23] Grivennikov SI, Greten FR, Karin M (2010). Immunity, inflammation, and cancer. Cell.

[24] Twito T, Chen Z, Khatri I, Wong K, Spaner D, Gorczynski R (2013). Ectodomain shedding of CD200 from the B-CLL cell surface is regulated by ADAM28 expression. Leukemia research.

[25] Hallek M, Cheson BD, Catovsky D, Caligaris-Cappio F, Dighiero G, Dohner H, Hillmen P, Keating MJ, Montserrat E, Rai KR, Kipps TJ (2008). Guidelines for the diagnosis and treatment of chronic lymphocytic leukemia: a report from the International Workshop on Chronic Lymphocytic Leukemia updating the National Cancer Institute-Working Group 1996 guidelines. Blood.

[26] Xu W, Li JY, Wu YJ, Yu H, Shen QD, Li L, Fan L, Qiu HX (2008). Prognostic significance of ATM, TP53 deletions in Chinese patients with chronic lymphocytic leukemia. Leukemia research.

[27] Hamblin TJ, Orchard JA, Ibbotson RE, Davis Z, Thomas PW, Stevenson FK, Oscier DG (2002). CD38 expression and immunoglobulin variable region mutations are independent prognostic variables in chronic lymphocytic leukemia, but CD38 expression may vary during the course of the disease. Blood.

